# The association of atherogenic index of plasma with cardiovascular outcomes in patients with coronary artery disease: A systematic review and meta-analysis

**DOI:** 10.1186/s12933-024-02198-y

**Published:** 2024-04-02

**Authors:** Mehrdad Rabiee Rad, Ghazal Ghasempour Dabaghi, Bahar Darouei, Reza Amani-Beni

**Affiliations:** 1 Interventional Cardiology Research Center, Cardiovascular Research Institute, Isfahan University of Medical Sciences, Isfahan, Iran; 2 School of Medicine, Isfahan University of Medical Science, Isfahan, Iran; 3 Isfahan Cardiovascular Research Center, Cardiovascular Research Institute, Isfahan University of Medical Sciences, Isfahan, Iran

**Keywords:** Atherogenic index of plasma, AIP, Coronary artery disease, CAD, Prognosis, Outcomes

## Abstract

**Background:**

Atherogenic index of plasma (AIP) represents a novel marker in the current era of cardiovascular diseases. In this meta-analysis, we aimed to evaluate the association of AIP with cardiovascular prognosis in patients with coronary artery disease (CAD).

**Methods:**

PubMed, Scopus, and Web of Science databases were searched from inception through 2024. The primary outcome was major cardiovascular events (MACE). The secondary outcomes included all-causes death, cardiovascular death, myocardial infarction (MI), stroke, revascularization, and no-reflow phenomenon. AIP was determined by taking the logarithm of the ratio of triglyceride (TG) to high-density lipoprotein cholesterol (HDL-C). The data analysis was represented using the risk ratio (RR) along with a 95% confidence interval (CI).

**Results:**

Sixteen studies with a total number of 20,833 patients met the eligible criteria. The pooled-analysis showed a significant increased risk of MACE in the highest AIP group compared with the lowest AIP group (RR = 1.63; 95% CI, 1.44–1.85; *P* < 0.001). A similar result was observed when AIP was regarded as a continuous variable (RR = 1.54; 95% CI, 1.30–1.83; *P* < 0.001). Besides, elevated AIP was associated with increased risk of cardiovascular death (RR = 1.79; 95% CI, 1.09–2.78; *P* = 0.02), MI (RR = 2.21; 95% CI, 1.55–3.13; *P* < 0.001), revascularization (RR = 1.62; 95% CI, 1.34–1.97; *P* < 0.001), no-reflow phenomenon (RR = 3.12 95% CI, 1.09–8.96; *P* = 0.034), and stent thrombosis (RR = 13.46; 95%CI, 1.39-129.02; *P* = 0.025). However, AIP was not significantly associated with the risk of all-causes death and stroke among patients with CAD.

**Conclusions:**

The results of this study demonstrated that increased AIP is an independent prognostic factors in patients with CAD. Further research is warranted to elucidate the potential development of targeted interventions to modify AIP levels and improve patient outcomes.

## Introduction

Coronary artery diseases (CAD) are accountable for a high morbidity and mortality rate worldwide, with 17.8 million deaths annually [1]. Many studies have been conducted on the role of risk factors in predicting the risk of CAD; however, fewer studies have addressed the role of various factors in the short and long-term prognosis of patients with CAD. The short-term prognosis is mainly related to percutaneous coronary intervention (PCI) and in-hospital events such as the no-reflow phenomenon and in-hospital death, while the long-term prognosis mainly includes major adverse cardiovascular events (MACE) [2].

The prognosis of patients with CAD is dependent upon multiple factors. Traditional and modifiable risk factors for CAD include hypertension, diabetes mellitus, smoking, obesity, and dyslipidemia, which have been identified to play a role in the prognosis of CAD and, therefore, MACE [3]. However, clinicians frequently come across patients with novel CADs that have been misclassified due to these traditional cardiovascular risk factors in a way that necessitates establishing accurate predictors for CAD [4].

Atherogenic index of plasma (AIP) is calculated by Logarithm [triglyceride (TG) / high-denisity lipoprotein cholesterol (HDL-C)] and can be an independent cardiovascular risk factor by correlating with lipoprotein particle size [5]. A recent meta-analysis concluded that higher values of AIP can significantly increase the risk of CAD after adjusting for other risk factors [6]. Moreover, other studies have revealed the prognostic role of AIP in arterial stiffness, atherosclerotic disease, the risk of AMI, ischemic stroke, and MACE [7–9]. Fu et al. demonstrated that diabetic patients with MACE had higher values of AIP, introducing a novel MACE predictor for high-risk patients [10]. Similar results were observed in another study, including non-diabetic older adults with hypertension [11]. Nevertheless, no meta-analysis has been performed to reveal AIP’s prognostic effect in patients with CAD; therefore, we sought to determine the association between the levels of AIP and prognosis in patients with CAD.

## Materials and methods

### Data sources and searches

This systematic review and meta-analysis is performed according to the guideline of the Preferred Reporting Items for Systematic Review and Meta-analyses statement (PRISMA) [12]. A systematic search of the electronic databases including PubMed, Scopus, and Web of Science was undertaken to identify relevant papers published before January 2024. Search strategy used the terms for AIP (“Atherogenic index of plasma”, “atherogenic index”, AIP) and CAD (“coronary disease”, “coronary diseases”, “disease coronary”, “coronary heart disease”, “coronary heart diseases”, “heart disease coronary”, “heart diseases coronary”, “left main”, “left main coronary disease”, “percutaneous coronary intervention”, “coronary artery disease”, “coronary artery diseases”, “coronary artery bypass”, “coronary artery bypass graft”, “coronary syndrome”, “acute coronary syndrome”, “chronic coronary syndrome”). We also conducted a manual search of reference lists and potential related articles. Two independent reviewers completed the electronic search in databases.

### Eligible criteria

Two reviewers independently screened the eligible studies based on the following inclusion criteria: (1) Adult patients who were diagnosed with CAD including myocardial infarction (MI), and acute or chronic CAD; (2) Measured AIP and reported the odds ratios (ORs) or hazard ratios (HRs) were with 95% confidence interval (CI) for association of AIP with the outcomes; and (3) The full text was available and written in the English language. Abstracts, reviews, case reports and case series, nonhuman studies, and letters to editors were excluded. Any disagreement was resolved by consensus.

### Data extraction and quality assessment

Major adverse cardiovascular events (MACE) was the primary outcome of interest. The secondary outcomes included all-causes mortality, cardiovascular mortality, MI, stroke, revascularization, and no-reflow phenomenon. The following information was abstracted by two independent investigators: the first author’s last name, publication date, sample size, country, study design, mean age, percent of female participants, type of CAD, length of follow-up, adjusted RRs with their 95% CI for the outcomes. Disagreements were resolved by a third reviewer.

Two reviewers independently conducted a quality assessment of each included study using the Newcastle–Ottawa Quality Assessment Scale (NOS), with scores of ≥ 7 considered as high-quality studies [13]. Any discrepancies were resolved through discussion.

### Statistical analyses

Risk ratios (RRs) and 95% CIs from the fully adjusted models were pooled to obtain the association of AIP with the outcomes. In studies where the AIP was examined as categorized variable, the RR of the outcomes for patients with the highest AIP level compared to those with the lowest level were collected. In studies where the AIP was analyzed as a continuous variable, the RR of the outcomes per 1-unit increase in the AIP were extracted.

The I^2^ statistic and Cochran’s Q test were utilized to assess heterogeneity. In cases where significant heterogeneity was observed (I^2^ > 50%, *p* < 0.1) among the studies, a random-effects model was applied. A fixed-effects model was used in case of no significant heterogeneity. Visual inspection of Funnel plot and Egger test were used to evaluate possible publication bias. We performed subgroup analysis to identify the potential sources of heterogeneities. All data were analyzed with STATA (Version14). P value < 0.05 was considered as significant.

## Results

**Fig. 1:**
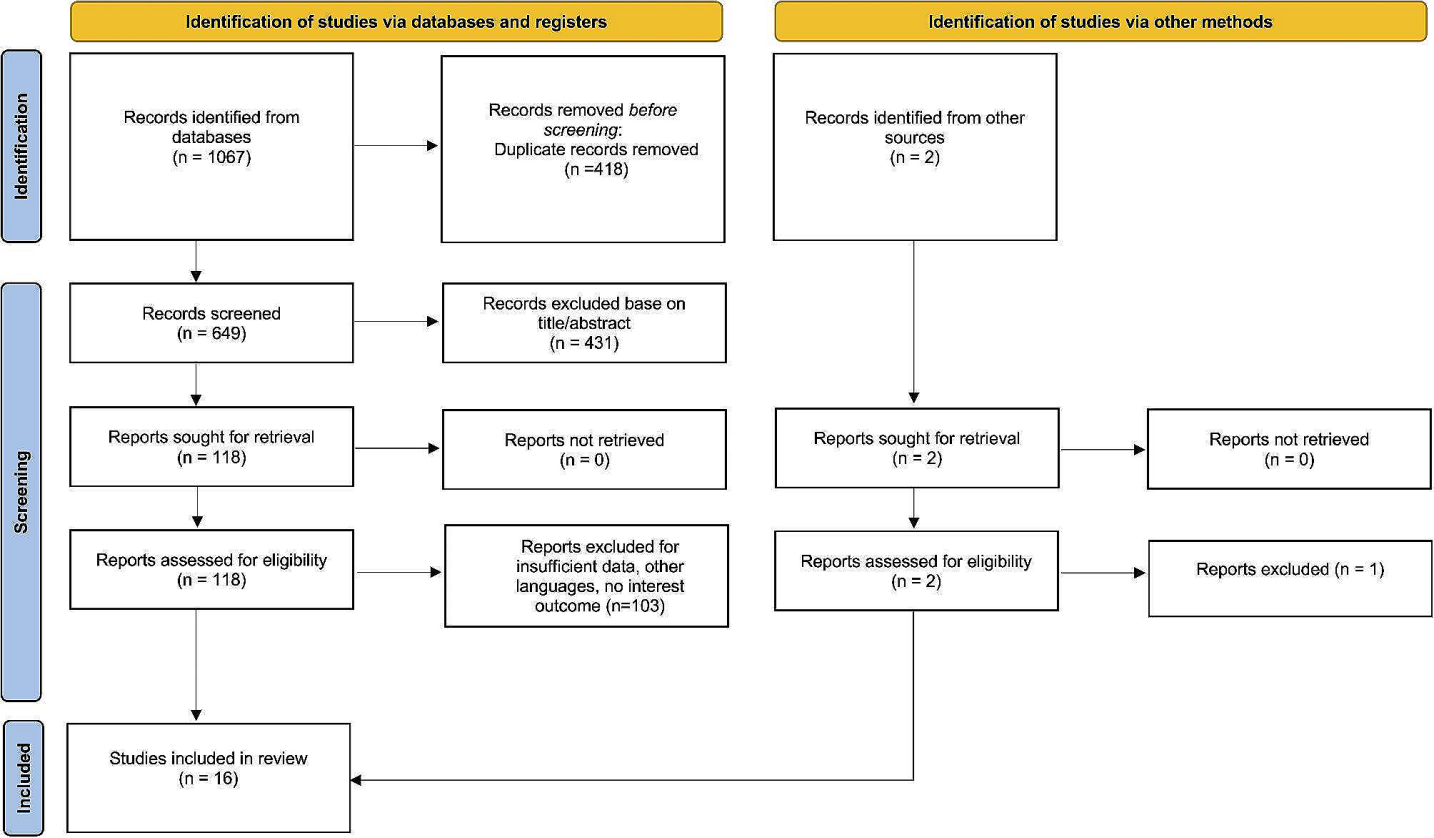
The flowchart of study selection

Following the abovementioned systematic search, we identified 1067 papers through databases. After duplicates removing and title/abstract screening, 118 studies eligible for full-text evaluation. After full-text screening, 16 studies [14–29] were included (Fig. 1).

### Characteristics of included studies

The basic characteristics of included studies are summarized in Table 1. Sixteen studies with a total of 20,883 participants were published from 2020 to 2024. Twelve studies were retrospective cohort, three studies were prospective cohort, and one study was cross-sectional. The mean age and female proportion ranged from 55 to 63 years and 14.7–41.1%, respectively. The duration of follow-up ranged from three day to four years. All included studies evaluated the AIP under fasting condition. According to NOS score, all included studies had high quality (score ≥ 7).

**Table 1:** Characteristics and findings of enrolled studies

First author	Year	Study design	Population	Sample size (n)	Age (mean ± SD)	Female (percent)	AIP (mean ± SD)	Follow-up (mean ± SD)	Main findings	MACE definition	NOS
Toprak et al.	2024	Retrospective cohort	Patients with STEMI who underwent primary PCI within 12 h	1284	58.80 ± 12.45	28.40%	0.64 ± 0.26	NR	AIP significantly increased the risk of no-reflow phenomenon (*p* < 0.001).		9
Wang et al.	2023	Retrospective cohort	Patients with ACS and LDL-C levels below 1.8mmol/L who underwent PCI.	1133	58.6 ± 9.5	14.70%	0.11	Median 26 month	AIP significantly increased the risk of MACCE (*p* = 0.026) and unplanned revascularization (*p* = 0.029) but did not significantly increase the risk of all-cause death (*p* = 0.494), cardiovascular death (*p* = 0.487), non-fatal MI (*p* = 0.114), and non-fatal stroke (*p* = 0.425).	MACE: cardiac death, non-fatal MI, non-fatal stroke, and unplanned repeat revascularization.	9
Liu et al.	2023	Retrospective cohort	Prediabetic patients with unstable angina pectoris	1096	59.47 ± 9.86	30.10%	0.06 ± 0.28	26.3 ± 6.5 month	AIP significantly increased the risk of the MACE (*p* < 0.001), non-fatal MI (*p* = 0.009), and refractory angina (*p* < 0.001) but did not significantly increase the risk of cardiac death (*p* = 0.460).	MACE: cardiac death, refractory angina, and non-fatal MI.	9
Erdoğan et al.	2023	Retrospective cohort	Patients with stable angina pectoris and/or angina-equivalent symptoms with intermediate risk in coronary computed tomography angiography with intermediate chronic coronary syndrome risk	715	55 [49–62]	42%	0.25 [0.12–0.38]	Median 17 months	AIP did not significantly increase the risk of MACE (*p* = 0.091).	MACE: non-fatal MI, hospitalization for heart failure, cerebrovascular events, non-cardiac mortality, and cardiac mortality.	9
Çelik et al.	2023	Retrospective cohort	Patients with ACS treated with PCI	848	59.93 ± 12.09	21.50%	0.50 ± 0.31	NR	AIP did not significantly increase the risk of no-reflow phenomenon (*p* = 0.422).		8
Alifu et al.	2023	Retrospective cohort	Patients with chronic coronary syndrome who underwent coronary angiography	404	63.61 ± 9.64	41.10%	0.15 ± 0.29	Median 35 months	AIP did not significantly increase the risk of MACE (*p* = 0.119).	MACE: cardiovascular death (deaths derived from heart failure, malignant arrhythmias, acute MI, or other cardiac conditions), Ischemia-driven revascularization, nonfatal MI, heart failure, and nonfatal stroke	9
Kasapkara et al.	2023	Retrospective cohort	Patients with STEMI who underwent primary PCI	873	59 [51–67]	19.20%	Non-survivor (53) = 0.59 [0.46–0.83] Survivors (820) = 0.47 [0.26–0.72]	Median 0.1 months	AIP significantly increased the risk of in hospital mortality (*p* = 0.012).		9
Özen et al.	2023	Retrospective cohort	Patients with ACS who underwent urgent coronary angiography	558	59 ± 18	24.37%	Median: 0.50	Median 12 months	AIP significantly increased the risk of MACE (*p* < 0.001).	MACE: cardiac death (death primarily due to acute MI, congestive heart failure, and malignant arrhythmia.), non-fatal MI, target vessel revascularization, congestive heart failure, and nonfatal stroke	8
Kan et al.	2023	Retrospective cohort	Patients with ACS who underwent either primary or elective PCI	1725	59.96 ± 10.37	23.30%		24 months	AIP significantly increased the risk of MACE (*P* < 0.001).	MACE: all-cause mortality, non-fatal ischemic stroke, non-fatal spontaneous myocardial infarction, and unplanned repeat revascularization	9
Abacıoğlu et al.	2022	Retrospective cohort	Patients with ACS who underwent PCI	698	63.3 ± 10.6	30.80%	0.24 ± 0.23	NR	AIP significantly increased the risk of stent thrombosis (*p* = 0.025).		8
Shao et al.	2022	Retrospective cohort	Patients with ACS who underwent primary or elective PCI	1694	60.0 ± 10.4	23.49%	0.15 ± 0.27	Median 30.9 months	AIP significantly increased the risk of MACE (*p* < 0.001).	MACE: all-cause mortality, non-fatal MI, non-fatal ischemic stroke, or unplanned repeat revascularization	9
Zheng et al.	2022	Prospective cohort	Patients with Non-diabetic CAD who underwent PCI	5538	57.41 ± 10.43	20.66%	0.18 ± 0.26	28 ± 2.3 months	AIP significantly increased the risk of MACE (*p* = 0.042), cardiac death/MI (*p* = 0.013), target vessel revascularization (*p* = 0.042), and MI (*p* = 0.004) but did not significantly increase the risk of all-cause death (*p* = 0.169), cardiac death (*p* = 0.828), and stroke (*p* = 0.973).	MACE: cardiac death, target vessel revascularization, and non-fatal MI	9
Refaat et al.	2021	Cross-sectional	Patients with acute STEMI who underwent primary PCI	400	60.31 ± 11.84	29%	0.58 ± 0.17	NR	AIP significantly increased the risk of no-reflow phenomenon (*p* = 0.04).		8
Süleymanoğlu et al.	2020	Retrospective cohort	patients with STEMI who underwent primary PCI	763	58 ± 12	15.07%	0.42 [0.29–0.53]	NR	AIP significantly increased the risk of no-reflow phenomenon (*p* < 0.001).		8
Qin et al.	2020	Prospective cohort	Patients with type 2 diabetes who underwent PCI	2356	57.97 ± 9.15	26.23%	0.24 ± 0.31	48 months	AIP significantly increased the risk of MACE (*p* = 0.011), all-cause death (*p* = 0.031), cardiac death (*p* = 0.011), cardiac death/MI (*p* < 0.001), MI (*p* = 0.001), Repeat revascularization (*p* < 0.001), target vessel revascularization (*p* < 0.001), and non-target vessel revascularization (*p* = 0.026) but did not significantly increase the risk of stroke (*p* = 0.694).	MACE: cardiogenic death, MI, repeated revascularization, and stroke.	9
Ma et al.	2020	Prospective cohort	Patients with type 2 diabetes and ACS who underwent PCI	798	61 ± 10	27.32%	0.26 ± 0.20	Median 30.9 months	AIP significantly increased MACE (*p* < 0.001) and secondary endpoint (*p* = 0.044).	MACE: all-cause mortality, non-fatal spontaneous MI, non-fatal ischemic stroke, and unplanned repeat revascularization. Secondary endpoint: cardiovascular death, non-fatal MI, and non-fatal ischemic stroke	9

AIP, atherogenic index of plasma; CAD, coronary artery disease; ACS, acute coronary syndrome; MACE, major adverse cardiovascular events; MI, myocardial infarction; PCI, percutaneous coronary intervention; STEMI, ST-elevation myocardial infarction; LDL-C, low density lipoprotein cholesterol; NR, not reported.

### Primary outcome

**Fig. 2:**
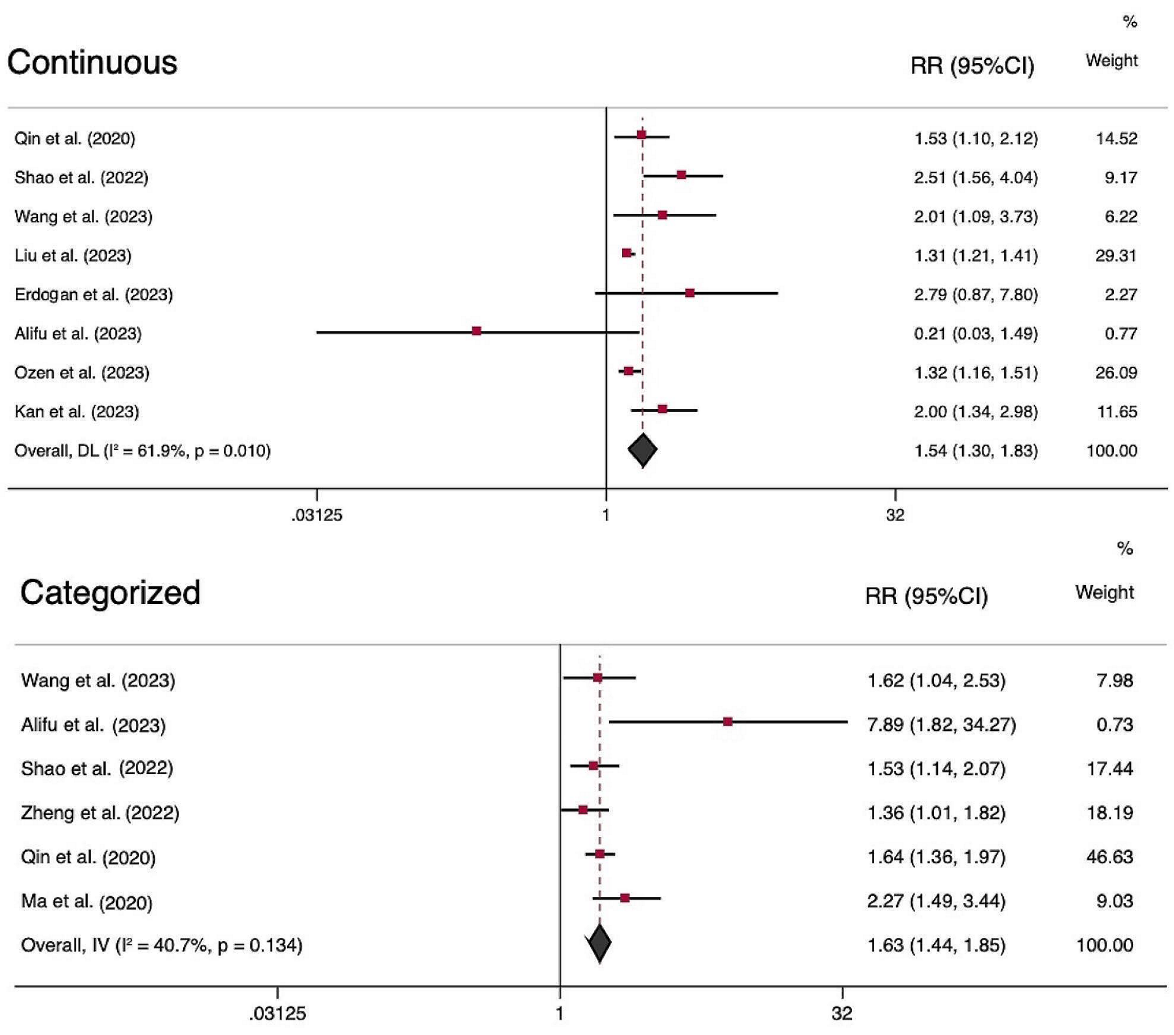
Forest plots showing the meta-analysis of major cardiovascular events using the AIP as categorial and continues variable

A total of eight studies investigated the association of AIP as a continues variable and MACE in patients with CAD. Overall, AIP level was found to increase the risk of MACE (RR = 1.54; 95% CI, 1.30–1.83; *P* < 0.001) with a significant heterogeneity (I^2^ = 61.9%, *P* = 0.010) (Fig. 2). Six studies compared the highest vs. lowest category of AIP, and the pooled analysis showed an increased risk of MACE in those with higher AIP (RR = 1.63; 95% CI, 1.44–1.85; *P* < 0.001) with no significant heterogeneity (I^2^ = 40.0%, *P* = 0.134) (Fig. 2).

### Secondary outcomes

**Fig. 3:**
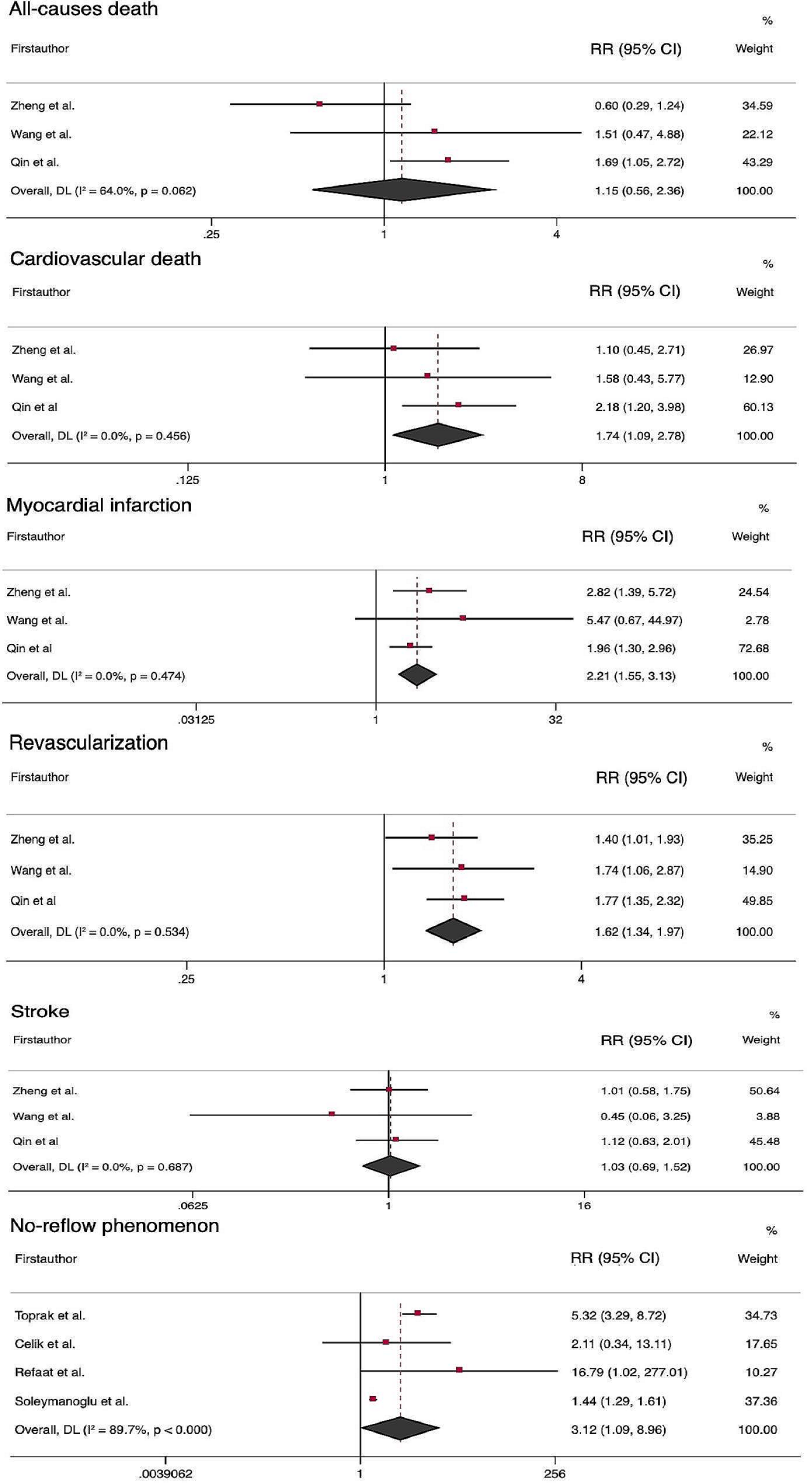
Forest plots showing the meta-analysis of secondary outcomes in comparison of highest AIP vs. lowest AIP group

Ten studies reported the RRs for the secondary outcomes. The pooled analysis indicated that higher AIP increase the risk of cardiovascular death (RR = 1.79; 95% CI, 1.09–2.78; *P* = 0.02), MI (RR = 2.21; 95% CI, 1.55–3.13; *P* < 0.001), revascularization (RR = 1.62; 95% CI, 1.34–1.97; *P* < 0.001), and no-reflow phenomenon (RR = 3.12 95% CI, 1.09–8.96; *P* = 0.034). However, AIP was not significantly associated with risk of all-causes death (RR = 1.15; 95% CI, 0.56–2.36; *P* = 0.699) and stroke (RR = 1.03; 95% CI, 0.69–1.52; *P* = 0.892) (Fig. 3). A significant heterogeneity was found for the no-reflow phenomenon (I^2^ = 89.7%, *P* < 0.001). Three studies analyzed AIP as a continuous variable, which reported an of HR 1.21 (95% CI, 0.72–2.02, *P* = 0.460), 1.61 (95% CI, 1.12–2.32, *P* = 0.009), 3.77 (95% CI, 1.34–10.60, *P* = 0.012), and 13.46 (95%CI, 1.39-129.02; *P* = 0.025) for cardiovascular death [17], MI [17], all-causes death [21], and stent thrombosis [24], respectively.

### Subgroup and sensitivity analysis

A subgroup analysis was performed for primary outcome according to the age (< 60 or ≥ 60 years), study design (retrospective or prospective), sample size (< 1,000 or ≥ 1,000), duration of follow-up (< 24 or ≥ 24 months), and LDL-C (< 1.8 or ≥ 1.8 mmol/L) to identify the sources of heterogeneity. A remarkable reduction in heterogeneity was found in prospective studies (I^2^ = 0.0%) and LCL-C below 1.8 mmol/L (I^2^ = 0.0%), suggesting that study design and LDL-C level might be factors contributing to heterogeneity. Besides, the analysis revealed no significant association between AIP and MACE in studies with a duration of follow-up below 24 months (RR = 1.56; 95% CI, 0.85, 2.87; *P* = 0.150) and mean age of over 60 years (RR = 1.43; 95% CI, 0.72, 2.82; *P* = 0.305) (Table 2).

**Table 2:** Subgroup analysis of the primary outcome

Subgroup	Studies (N)	Participants (N)	RR (95% CI)	P-value	I-squared (%)
**Age**					
≥ 60	3	2,656	1.43 (0.72, 2.82)	0.305	80.1
< 60	5	7,025	1.59 (1.26, 2.00)	< 0.001	51.6
**Study design**					
Retrospective	7	7,325	1.56 (1.28, 1.90)	< 0.001	66.4
Prospective	1	2,356	1.53 (1.10, 2.12)	0.011	0.0
**Sample size**					
≥ 1,000	5	8,004	1.71 (1.31, 2.23)	< 0.001	69.4
< 1,000	3	1,677	1.25 (0.51, 3.07)	0.628	61.6
**Duration of follow-up**					
≥ 24	6	8,408	1.66 (1.25, 2.20)	0.001	69.9
< 24	2	1,273	1.56 (0.85, 2.87)	0.150	43.0
**LDL-C**					
≥ 1.8	7	8,348	1.51 (1.27–1.81)	< 0.001	64.3
< 1.8	1	1,133	2.01 (1.09–3.72)	0.026	0.0

RR, relative risk; LDL-C, low density lipoprotein cholesterol.

A sensitivity analyses was performed including studies with a ≥ 2 years of follow-up. Consistent with our primary analysis, we revealed a significant association of AIP with MACE (RR = 1.66; 95% CI, 1.25, 2.20, *P* = 0.001). The results for other outcomes remained unchanged except for no-reflow phenomenon, which all studies reported a short duration of follow-up, and hence, the further analysis could not perform.

### Publication bias

**Fig. 4:**
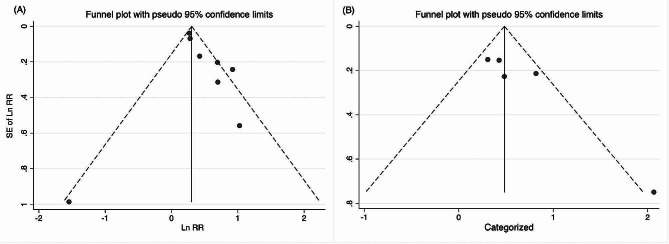
The publication bias assessment with funnel plot for the primary outcome

The funnel plots in Fig. 4 demonstrate the relationship between the AIP and the incidence of MACEs in CAD patients. Upon visual examination, the plots seem to be asymmetrical, suggesting a possible risk of publication bias. However, Egger test found no significant publication bias for categorial (*P* = 0.052) and continues (*P* = 0.178) analysis.

## Discussion

This meta-analysis showed that a higher AIP is associated with an increased risk of MACE, cardiovascular mortality, MI, revascularization, and the no-reflow phenomenon in patients with CAD. Subgroup analysis revealed that AIP may not be an indicator of MACE among patients aged ≥ 60 years and short follow-up times. Besides, AIP was not associated with all-causes mortality and stroke risk.

In this study, CAD patients with a higher AIP level had a < 1.5-fold higher risk for MACE compared with subjects with lower AIP. In line with our results, a fifteen-year cohort study conducted on 6323 healthy adults demonstrated a 1.2-fold greater risk for cardiovascular events among participants with higher AIP [30]. Moreover, a cross-sectional study compromising 7,362 adults showed that the third tertile of AIP had a 1.3-fold higher risk for cardiovascular disease compared to the first tertile [31]. These collective findings underscore the potential of AIP as a valuable biomarker for identifying individuals at higher risk for cardiovascular disease.

Our subgroup analysis did not detect a significant association between AIP and MACE in older patients. In this context, Nansseu et al. [32] enrolled 108 postmenopausal women, and found no significant correlation between AIP and cardiovascular risk evaluated with Framingham risk score. Similarly, there was no significant association between AIP and CAD in elderly females aged ≥ 65 years [33]. Moreover, AIP could not predict the presence of CAD in elderly males who underwent coronary angiography [34]. A possible explanation to address this finding is that AIP level is increased in elderly population [35]. Several studies showed a positive correlation between AIP and age among different populations [36–38], which might impact the likelihood of detecting a significant association between AIP level and cardiovascular events in this group. Further studies are warranted to better understand the impact of age on AIP levels and its implications for assessing cardiovascular risk in the elderly.

This meta-analysis included three studies evaluating the association of AIP as a categorial variable with all-causes death, and the results showed that AIP could not predict all-causes death in patients with CAD. However, Refs. [16, 26, 29]) considered AIP as a continuous variable, and found a significant positive correlation between AIP level and risk of all-causes mortality [21]. This discrepancy may be due to low number of studies and the differences in follow-up duration between the studies. The endpoint of et al. was the in-hospital mortality, and hence, their results showed the predictive value of AIP for all-causes mortality in a median follow-up duration three days. To evaluate the association of AIP with risk of short-term mortality, further studies are needed.

The disbalance of these plasma lipids leads to dyslipidemia, which is characterized by high levels of LDL-C, TG, and total cholesterol and low levels of HDL-C [39]. Although reducing LDL-C levels is a treatment goal in CAD, even after attaining this target, a notable residual cardiovascular risk remains present, encouraging the exploration of more accurate risk factors in these patients [40]. Regarding a practical predictor, the AIP strongly predicts cardiovascular events by reflecting the atherogenic lipid profile and providing valuable insights into the residual cardiovascular risk.

Despite the strengths of this study, including a comprehensive search strategy and evaluating several outcomes, there are limitations that should be considered. First, the majority of studies included in this meta-analysis were retrospective, and hence, further studies with prospective design are needed. Second, the presence of significant heterogeneity in some of the analyses suggests potential variations in methodologies and outcome definitions such as MACE, which may have influenced the results. Third, although this study found a significant association of AIP with MI and cardiovascular death, there was considerable variability across studies. As such, additional validation is required to confirm this tenuous relationship. Finally, the included studies were observational, which limits the ability to establish causal relationships between AIP and cardiovascular outcomes.

## Conclusion

In conclusion, the findings of this meta-analysis support the notion that AIP is a potential prognostic marker for adverse cardiovascular events in patients with CAD. A higher AIP was consistently associated with an increased risk of MACE, cardiovascular death, MI, revascularization, and the no-reflow phenomenon. Notably, no association was found between AIP and all-causes death or stroke. These results have important implications for risk stratification and management strategies in CAD patients. Further research is needed to validate these findings.

## Data Availability

No datasets were generated or analysed during the current study.

## Abbreviations

***AIP***: Atherogenic index of plasma
***CAD***: Coronary artery disease
***HDL-C***: High-density lipoprotein cholesterol
***HR***: Hazard ratio
***LDL-C***: Low-density lipoprotein cholesterol
***OR***: Odds ratio
***PCI***: Percutaneous coronary intervention
***PRISMA***: Preferred Reporting Items for Systematic Review and Meta-analyses statement
***RR***: Risk ratio
***sdLDL***: Small dense low-density lipoprotein
***TG***: Triglyceride
